# Surgical Outcome and Evaluation of Strategies in the Management of Growth Hormone-Secreting Pituitary Adenomas After Initial Transsphenoidal Pituitary Adenectomy Failure

**DOI:** 10.3389/fendo.2022.756855

**Published:** 2022-04-14

**Authors:** Jiun-Lin Yan, Mao-Yu Chen, Yao-Liang Chen, Chi-Cheng Chuang, Peng-Wei Hsu, Kuo-Chen Wei, Chen-Nen Chang

**Affiliations:** ^1^ Department of Neurosurgery, Keelung Chang Gung Memorial Hospital of the Chang Gung Medical Foundation, Keelung, Taiwan; ^2^ Chang Gung University, College of Medicine, Taoyuan, Taiwan; ^3^ Department of Radiology, Keelung Chang Gung Memorial Hospital of the Chang Gung Medical Foundation, Keelung, Taiwan; ^4^ Department of Neurosurgery, Chang Gung Memorial Hospital Linkou Main Branch, Taoyuan, Taiwan; ^5^ Department of Neurosurgery, Xiamen Chang Gung Hospital, Xiamen, Taiwan

**Keywords:** acromegaly, growth hormone, pituitary adenoma, transsphenoidal pituitary adenectomy, octreotide

## Abstract

Acromegaly is a systemic disease that requires multidisciplinary treatment to achieve the best clinical outcome. This study aimed to evaluate the outcomes of the endoscopic transsphenoidal approach (TSA) as the primary treatment for somatotroph adenomas and further investigate patients who had suboptimal surgical results. This retrospective study included 83 patients with somatotroph adenomas treated by TSA at our institution from 1999 to 2010. Biochemical remission was defined as hGH <1 and <2.5 ng/ml. Factors associated with failure of TSA and strategy of secondary treatments for refractory and recurrent disease were analyzed. The mean age of patients was 41.1 ± 11.3 years, and the mean follow-up time was 54.2 ± 44.3 months. Approximately 44.5% of patients had residual tumors after TSA. Larger tumor size, higher GH level before the operation, and the existence of residual tumors were associated with TSA failure. Forty-one patients had an inadequate response to TSA or a recurrent lesion, and of these patients, 37 had residual tumor after TSA. Octreotide results in good outcomes in the treatment of DGSA patients, and SRS/EXRT generates good results in treating patients who receive second treatments when remission cannot be reached 6 months after TSA operation.

## Introduction

The pituitary gland can be considered the master hormone gland because it regulates most of the body’s hormone production, which in turn regulates overall metabolism (1). Pituitary adenomas account for approximately 16% of intracranial tumors (2, 3). Although most pituitary adenomas are small in size and do not produce specific symptoms, they can result in marked disturbances of hormone regulation and symptoms such as severe headaches and visual field disturbances due to the tumor mass effect (2, 3).

Somatotroph adenomas account for approximately 20% of pituitary tumors (4, 5). Growth hormone (GH) is secreted by somatotroph cells in the anterior pituitary (2, 3, 6, 7). Upon binding to the GH receptor in the liver, GH initiates the production of insulin-like growth factor (IGF)-1, which affects bone and tissue growth and carbohydrate, protein, and lipid metabolism (6–8). This can further lead to acromegaly which is classically presented with symptoms of enlarged hands and feet (7, 9). In addition, persistent GH excess is associated with increased morbidity and mortality due to systemic effects in the heart, liver, colon, and endocrine system (4, 6, 7, 9). Therefore, aggressive hormone control is the goal for acromegalic patients.

Based on the pattern of hormone and transcription factor production, somatotroph adenoma can be divided into four subtypes. They are densely granulated somatotroph adenoma (DGSA), sparsely granulated somatotroph adenoma (SGSA), mammosomatotrophs, and mixed somatotroph–lactotroph tumors (10, 11). DGSA is presented in 30%–50% of acromegaly cases (10, 12). The cells are eosinophilic and highly express GH and α-subunits. DGSA usually grows slowly, is preferred in older patients, and shows an excellent response to treatment with somatostatin ligands or analogs. On the other hand, SGSA accounts for another 15%–35% of acromegaly patients (10, 12). The cells are lightly eosinophilic or chromophobic. SGSA usually demonstrates focal or weak GH expression and no α-subunit expression. SGSA is usually more aggressive, rapid growing, and common at a younger age.

Although radiosurgery techniques and chemotherapy have been refined in the past decades, surgical removal is the primary treatment for somatotroph adenomas (4, 13–16). In particular, the endoscopic transsphenoidal approach (TSA) for pituitary tumor resection has been regarded as the initial treatment for pituitary adenomas since the 1990s (3, 4, 13, 15). TSA appears to provide better outcomes and is associated with fewer complications than craniotomy (5, 17). However, primary TSA is not always successful. After surgery, the biochemical control rate was about 50%–60%, and aggressive pituitary adenomas often require multimodal therapy (2, 14, 16, 18). In multimodal therapy, radiosurgery and medical therapy are involved. These therapies generally represent second-line options and are advised when remission is not achieved after TSA operation and in patients with recurrent or refractory pituitary adenomas (18–21).

Medical therapy of acromegaly includes taking somatostatin receptor ligands, cabergoline, and pegvisomant (12). Somatostatin receptor ligands are the most widely used drug for acromegaly. They activate somatostatin receptors, thereby inhibiting GH secretion, suppressing proliferation, and promoting apoptosis (22). Pegvisomant is a GH analog that binds to GH receptors with high affinity, but without activating the downstream signal pathway (23). Cabergoline is an off-label used dopamine receptor agonist (24). Cabergoline and pegvisomant are usually used with somatostatin receptor ligands together to achieve better results.

This study aims to evaluate the outcomes of TSA as the primary treatment for GH-secreting pituitary adenomas and to examine the responses of secondary treatments in patients who failed primary TSA operation and those with recurrent or refractory adenomas.

## Materials and Methods

All acromegalic patients were evaluated and treated by an acromegalic team composed of neurosurgeons, endocrinologists, radiologists, and radiation oncologists at our institution from 1999 to 2010. The records of patients with somatotroph adenomas treated by mono-nostril endoscopic transsphenoidal surgery, one kind of TSA, were retrospectively reviewed. This study was approved by the Institutional Review Board of our hospital (201901259B0), and because of the retrospective nature of the study, the requirement of informed patient consent was waived.

The inclusion criteria were (1) somatotroph adenoma; (2) primary treatment with TSA; and (3) follow-up for more than 1 year. Patients were excluded if the primary treatment was a transcranial pituitary adenectomy and if follow-up was less than 1 year.

### Radiological Measurement of Tumor Size

All patients received preoperative magnetic resonance imaging (MRI) of the brain with a 1.5- or 3.0-Tesla system (GE Company, Fairfield, Connecticut, USA; Siemens, Munich, Germany; Philips, Amsterdam, Netherlands). Sagittal, coronal, and axial MR images, including T1- and T2-weighted imaging, were acquired for all patients. T1-weighted sequences were performed after intravenous injection of gadolinium at a dose of 0.1 mmol/kg. The diameter of the pituitary tumor was measured, and tumors were classified by size as microadenoma (<1 cm), macroadenoma (1–2 cm), or giant pituitary adenoma (>2 cm). The Knosp classification was used to evaluate parasellar extension and cavernous sinus invasion of the pituitary tumor on preoperative MR images (25). Immediate postoperative, 3-month postoperative, and long-term follow-up MRI were performed to evaluate the existence of residual or recurrent tumors.

### Biochemical Remission

Serum GH levels were measured by an immunoradiometric assay. A random GH level, also known as basal GH, was measured in the morning with patients fasting without any glucose load. All GH assays for the same patient were performed in the same manner throughout the follow-up period. Since GH level was measured without any glucose load, the expression level of IGF-1 was under full control of GH and was not included in our study.

The remission criteria are a random GH <2.5 or <1.0 ng/ml at 6 months after TSA as other studies (26–28). Based on whether patients achieved remission at different time points postoperatively, they were categorized into remission or nonremission groups.

### Secondary Treatment After Initial Treatment Failure

During the follow-up, the GH level was measured every 3 months. Tumor recurrence or refractory condition was defined as a nonremission GH level by 6 months after surgery. Patients who did not achieve remission were either observed or received secondary treatment. Secondary treatments included second TSA operation, 20 mg (maximum quantity) octreotide daily, TSA operation plus octreotide, Novalis stereotactic radiosurgery, or observation.

### Statistical Analysis

Data extracted from the medical records included patient demographic information, GH level at diagnosis, preoperative GH level, tumor size, response to treatment, follow-up GH levels, and management of recurrence or inadequate response to the primary TSA operation.

Continuous data with normal distribution were presented as mean ± standard deviation and performed by unpaired *t*-test. Data without normal distribution were presented as median and interquartile range (IQR) and performed by Wilcoxon rank-sum test. Categorical data were presented as numbers (percentage) and performed by the Chi-square or Fisher’s exact test, as appropriate. Variables including age, sex, tumor size, GH level at diagnosis, GH level at preoperation, residual tumor, and histology subtypes were analyzed with univariate and multivariate analyses. In the multivariate, Cox regression models were used to estimate the hazard ratio (HR) and 95% confidence intervals (CIs) of the probability for achieving GH <1 and <2.5 ng/ml. The time to achieving a fasting GH level <1 or <2.5 ng/ml was drawn as a cumulative incidence function (CIF) using the Kaplan–Meier method. Statistical analyses were performed with SAS version 9.4 software (SAS Institute, Inc.), and data were graphed using GraphPad Prism (GraphPad Software Inc., California, USA). Values of *p* < 0.05 were considered to indicate statistical significance.

## Results

### Patients

As shown in **Table 1**, 83 patients (39 males and 44 females) with somatotroph adenomas were included in the study. The mean age was 41.1 ± 11.3 years, and the mean following time was 54.2 ± 44.3 months. Of these patients, 17 of them had microadenoma, 30 of them had macroadenoma, and 31 had giant pituitary adenoma.

**Table 1:** Patient characteristics (*N* = 83).

Variable	*N* (%) or mean ± SD
Sex	
Male	39 (46.9)
Female	44 (53.0)
Age at diagnosis (years)	41.1 ± 11.3
Follow-up (months)	54.2 ± 44.3
Tumor size	
Microadenoma (≤1 cm)	17 (21.8)
Macroadenoma (1–2 cm)	30 (38.5)
Giant pituitary adenoma (>2 cm)	31 (39.7)
Missing	5
GH level at diagnosis (ng/ml)^a^	37.8 ± 49.9
GH level preoperatively (ng/ml)^a^	40.5 ± 63.5
Biochemical response	
Time to GH <1 ng/ml	39 (47.0), 12.5 ± 20.3
Time to GH <2.5 ng/ml	60 (72.3), 11.3 ± 22.7
Histology subtypes	
DGSA	57 (72.2)
SGSA	22 (27.8)
Unrecognizable	4
After first operation	
Residual tumor	37 (44.6)
Received second operation	19 (22.9)
Received octreotide	26 (31.3)

a Two patients missing the information.

SD, standard deviation; DGSA, densely granulated somatotroph adenoma; SGSA, sparsely granulated somatotroph adenoma.

Histologically, 57 patients were categorized to DGSA, and the other 22 patients were SGSA. After the first TSA operation, 37 patients (44.6%) had a residual tumor, 19 patients (22.9%) received a secondary TSA operation, and 26 patients (31.3%) received medical treatment after the first TSA operation.

### Mortality and Morbidity

Three patients died after the primary TSA operation. Two deaths were due to a central nervous system (CNS) infection with sepsis, and the others were due to upper gastrointestinal (UGI) bleeding. Morbidities after the first TSA operation included 24 cases of transient diabetes insipidus (DI) (resolved in 3 months), 3 CNS infections, 10 cases of hypogonadism, 4 cases of adrenal insufficiency, 4 cases of hypothyroidism, 7 cases of cerebrospinal fluid leakage, 3 cases of sinusitis, 3 cases of panhypopituitarism, and 1 patient who developed seizures (**Table 2**).

**Table 2:** Mortality and morbidity through all pateints after first operation.

Variable	*N* (%)	
**Mortality**		
CNS infection with sepsis	2 (2.4)	
UGI bleeding	1 (1.2)	
**Morbidity**		
DI	24 (28.9)	Mostly transient, and resolved in 3 months
CNS infection	3 (3.6)	
Hypogonadism	10 (12.0)	
Adrenal insufficiency	4 (4.8)	
Hypothyroidism	4 (4.8)	
CSF leakage	7 (8.4)	
Sinusitis	3 (3.6)	
Panhypopituitarism^a^	3 (3.6)	
Seizure	1 (1.2)	

CNS, central nervous system; CSF, cerebrospinal fluid; DI, diabetes insipidus, UGI, upper gastrointestinal.

a Panhypopituitarism defined as hypofunction of >2 hormones.

### Biochemical Remission

**Figure 1:**
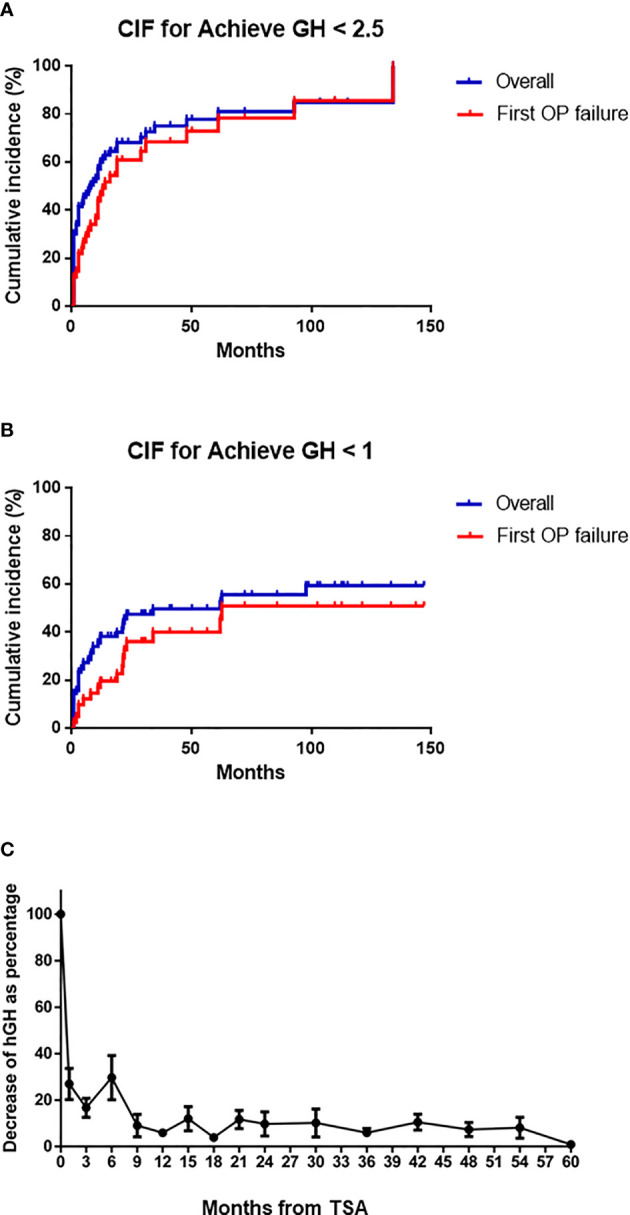
Cumulative incidence function (CIF) for achieved biochemical remission in the whole group and patients with first surgery failure. **(A)** GH <1 ng/ml. **(B)** GH <2.5 ng/ml. **(C)** The decreased percentage of hGH level in the whole group at different time points.

The figure of CIF shows about 40% and 83% of overall patients achieved GH level <2.5 ng/ml at 6 and 100 months after the operation, respectively (**Figure 1A**). For patients who failed the first TSA operation, the ratio of remission at 6 and 100 months are 22% and 83%, respectively. In a more stringent remission standard GH level <1 ng/ml, 28% and 59% of all patients achieved GH level <1 ng/m at 6 and 100 months, respectively. However, only 12% and 49% of patients who failed the first TSA operation achieved this criterion at 6 and 100 months, respectively (**Figure 1B**). As shown in **Figure 1C**, the maximum decrease in GH occurred during the first 3 months after TSA surgery.

### The Effect Factors on Achieving Biochemical Remission

**Table 3** shows the factors associated with remission in univariate and multivariate analyses at 6 months after first TSA surgery. In the univariate analysis, GH level at diagnosis, GH level at preoperation, tumor size, and residual tumor all had a significant association with the remission results. Patients with lower GH levels at diagnosis and preoperation, smaller tumor size, and had no residual tumor had more proportion to achieve biochemical remission at GH <1 or <2.5 ng/ml. Patients with DGSA histology had more proportion to achieve GH <2.5 ng/ml than those with SGSA (52.6% vs. 27.3%, *p* value = 0.042). In the multivariate, no residual tumor is the only factor that has a significant effect for achieving GH <1 ng/ml (HR = 7.20, 95% CI = 1.36–38.1).

**Table 3:** Factors associated with remission at 6 months after first surgery in univariate and multivariate analyses.

Variables	Achieve GH <1 ng/ml					Achieve GH <2.5 ng/ml				
	Univariate			Multivariate		Univariate			Multivariate	
	Yes (*n* = 22)	No (*n* = 61)	*p*-value	HR (95% CI)	*p*-value	Yes (*n* = 38)	No (*n* = 45)	*p*-value	HR (95% CI)	*p*-value
**Age**	43.2 ± 8.8	40.4 ± 12.1	0.317	1.00 (0.96–1.05)	0.833	43.4 ± 9.8	39.3 ± 12.2	0.100	1.01 (0.97–1.04)	0.691
**Sex**			0.407					0.613		
**Female**	10 (22.7)	34 (77.3)		ref		19 (43.2)	25 (56.8)		ref	
**Male**	12 (30.8)	27 (69.2)		1.06 (0.42–2.69)	0.897	19 (48.7)	20 (51.3)		0.96 (0.48–1.94)	0.92
**GH level at diagnosis**	10.4 (7.2–30.7)	27.6 (14.1–50.0)	**0.006**			13.6 (6.7–32.1)	33.8 (17.2–56.4)	**<0.001**		
**GH level at preoperation**	10.4 (7.2–23.9)	31.7 (14.1–50.0)	**0.002**	0.98 (0.96–1.01)	0.248	12.1 (6.0–33.2)	32.3 (17.5–52.0)	**<0.001**	0.98 (0.96–1)	0.054
**Tumor size >1 cm**			0.012^a^					**0.022**		
**Yes**	12 (19.7)	49 (80.3)		–		24 (39.3)	37 (60.7)		–	
**No**	9 (52.9)	8 (47.1)		–		12 (70.6)	5 (29.4)		–	
**Missing**	1	4				2	3			
**Tumor size >2 cm**			**0.023**					**0.014**		
**Yes**	4 (12.9)	27 (87.1)		ref		9 (29.0)	22 (71.0)		ref	
**No**	17 (36.2)	30 (63.8)		0.55 (0.13–2.31)	0.415	27 (57.4)	20 (42.6)		0.82 (0.28–2.42)	0.722
**Missing**	1	4				2	3			
**Residual tumor**			**<0.001**					**<0.001**		
**Yes**	2 (5.4)	35 (94.6)		ref		9 (24.3)	28 (75.7)		ref	
**No**	20 (43.5)	26 (56.5)		7.20 (1.36–38.1)	**0.020**	29 (63.0)	17 (37.0)		2.31 (0.9–5.89)	0.081
**Histology subtypes**			0.106					**0.042**		
**DGSA**	18 (31.6)	39 (68.4)		1.68 (0.44–6.35)	0.446	30 (52.6)	27 (47.4)		1.81 (0.67–4.89)	0.243
**SGSA**	3 (13.6)	19 (86.4)		ref		6 (27.3)	16 (72.7)		ref	
**Missing**	1	3				2	2			

GH, growth hormone; DGSA, densely granulated somatotroph adenoma; SGSA, sparsely granulated somatotroph adenoma. Significant results are shown in bold.

Continous data with normal distribution are presented as mean ± SD and performed by Student’s t-test; continuous data without normal distribution are presented as median (IQR) and performed by Wilcoxon rank sum test; categorical data are presented as n (%) and performed by Chi-squared test or Fisher’s exact test^a^, as appropriate.

### Stratified Analysis of Factors Associated With Residual Tumor

Further analysis of the risk factors of the residual tumor after first TSA showed that higher GH level at diagnosis (*p* = 0.003), presurgical tumor size (>2 cm, *p* < 0.001; >1 cm, *p* = 0.002), with DI syndrome (*p* = 0.036), and had histology subtype of SGSA (*p* = 0.005) were all significantly correlated with residual tumor (**Table 4**).

**Table 4:** Association of residual tumor and other variables.

Variable	Residual tumor		*p*-value
	Yes (*n* = 37)	No (*n* = 46)	
**GH level at diagnosis**	31.6 (18.4–68.2)	14.1 (8.7–34.6)	**0.003**
**Tumor size >1 cm**			**0.002**
**Yes**	33 (54.1)	28 (45.9)	
**No**	2 (11.8)	15 (88.2)	
**Missing**	2	3	
**Tumor size >2 cm**			**<0.001**
**Yes**	26 (83.9)	5 (16.1)	
**No**	9 (19.2)	38 (80.9)	
**Missing**	2	3	
**DI**			**0.036**
**Yes**	15 (62.5)	9 (37.5)	
**No**	22 (37.2)	37 (62.7)	
**Histology subtypes**			**0.005**
**DGSA**	19 (33.3)	38 (66.7)	
**SGSA**	15 (68.2)	7 (31.8)	
**Missing**	3	1	

GH, growth hormone; DGSA, densely granulated somatotroph adenoma; SGSA, sparsely granulated somatotroph adenoma. Significant results are shown in bold.

Continuous data are presented as median (IQR) and performed by Wilcoxon rank sum test; categorical data are presented as n (%) and performed by Chi-squared test.

### Factors Associated With Remission in Patients Failed First TSA Operation

Further analysis of the factors associated with remission at the final follow-up time of patients who failed the first TSA operation is shown in **Table 5**. A total of 41 patients had an inadequate response after the first TSA. Also, of these patients, 27 had a residual tumor. In the univariate analysis, age at diagnosis and histology subtypes had a significant association with the remission results. After adjusted for other covariates, sex (male vs. female patients: HR = 5.70, 95% CI = 1.06–30.56, *p* = 0.042), tumor size (≤2 vs. >2 cm: HR = 6.85, 95% CI = 1.11–42.13, *p*=0.038), and treatment (SRS/EXRT only vs. combine: HR = 29.82, 95% CI = 1.17–760.52, *p* = 0.040) had significant effect for achieving GH <1 ng/ml. For achieving GH <2.5 ng/ml, age is the only factor with significance.

**Table 5:** Factors associated with remission at final follow-up time for patients failed first TSA operation in univariate and multivariate analyses (*n* = 41).

Variables	Achieve GH <1 ng/ml					Achieve GH <2.5 ng/ml				
	Univariate			Multivariate		Univariate			Multivariate	
	Yes (*n* = 16)	No (*n* = 25)	*p*-value	HR (95% CI)	*p*-value	Yes (*n* = 30)	No (*n* = 11)	*p*-value	HR (95% CI)	*p*-value
**Age**	43.8 ± 9.7	36.8 ± 10.6	**0.040**	1.06 (0.98–1.15)	0.127	41.5 ± 10.7	34.0 ± 8.9	**0.046**	1.06 (1.00–1.12)	**0.046**
**Sex**			0.812					0.309^a^		
**Female**	9 (37.5)	15 (62.5)		ref		16 (66.7)	8 (33.3)		ref	
**Male**	7 (41.2)	10 (58.8)		5.70 (1.06–30.56)	**0.042**	14 (82.4)	3 (17.6)		2.71 (0.84–8.68)	0.094
**GH level at diagnosis**	31.4 (9.1–53.2)	28.5 (17.7–74.2)	0.659	–		27.6 (9.8–50.0)	33.9 (27.0–75.0)	0.231	–	
**GH level at preoperation**	26.4 (9.1–50.8)	30.5 (17.7–62.7)	0.553	1.00 (0.99–1.01)	0.828	29.2 (9.5–45.2)	33.9 (27.0–73.4)	0.262	0.99 (0.98–1)	0.236
**Tumor size >1 cm**			0.609^a^					0.309^a^		
**Yes**	12 (34.3)	23 (65.7)		–		24 (68.6)	11 (31.4)		–	
**No**	2 (50.0)	2 (50.0)		–		4 (100.0)	0 (0.0)		–	
**Missing**						3	2			
**Tumor size >2 cm**			0.051					0.073^a^		
**Yes**	5 (22.7)	17 (77.3)		ref		13 (59.1)	9 (40.9)		ref	
**No**	9 (52.9)	8 (47.1)		6.85 (1.11–42.13)	**0.038**	15 (88.2)	2 (11.8)		1.77 (0.55–5.74)	0.341
**Missing**						3	2			
**Residual tumor**			0.087					**0.007^a^**		
**Yes**	8 (29.6)	19 (70.4)		ref		16 (59.3)	11 (40.7)		ref	
**No**	8 (57.1)	6 (42.9)		0.52 (0.12–2.34)	0.396	14 (100.0)	0 (0.0)		1.13 (0.35–3.67)	0.833
**Histology subtypes**			**0.047**					**0.030^a^**		
**DGSA**	12 (52.2)	11 (47.8)		0.81 (0.15–4.31)	0.801	20 (87.0)	3 (13.0)		1.13 (0.30–4.23)	0.857
**SGSA**	3 (20.0)	12 (80.0)		ref		8 (53.3)	7 (46.7)		ref	
**Missing**										
**Treatment**			0.436^a^					0.165 ^a^		
**Second surgery only**	4 (50.0)	4 (50.0)		4.60 (0.27–78.95)	0.293	5 (62.5)	3 (37.5)		1.04 (0.17–6.50)	0.967
**Octreotide only**	8 (44.4)	10 (55.6)		3.22 (0.17–60.4)	0.434	16 (88.9)	2 (11.1)		0.71 (0.12–4.29)	0.710
**SRS/EXRT only**	2 (50.0)	2 (50.0)		29.82 (1.17–760.52)	**0.040**	3 (75.0)	1 (25.0)		1.28 (0.15–10.63)	0.817
**Combine treatment**	2 (18.2)	9 (81.8)		ref		6 (54.5)	5 (45.5)		ref	

GH, growth hormone; DGSA, densely granulated somatotroph adenoma; SGSA, sparsely granulated somatotroph adenoma. Significant results are shown in bold.

Continous data with normal distribution are presented as mean ± SD and performed by Student’s t-test; continuous data without normal distribution are presented as median (IQR) and performed by Wilcoxon rank sum test; categorical data are presented as n (%) and performed by Chi-squared test or Fisher’s exact test^a^, as appropriate.

We further investigated the association between histology subtypes and final remission after different treatments for patients who failed the first TSA operation (**Table 6**). Of the 41 patients, 8 received a second operation, 18 were treated with octreotide 20 mg, 4 received stereotactic radiosurgery (SRS) or conventional external beam radiotherapy (EXRT), and 11 received combined treatment. Patients received octreotide with DSGA had significant higher proportion than those with SGSA to reach GH <2.5 ng/ml (100% vs. 33.3%, *p* = 0.025).

**Table 6:** The association between histology subtypes and remission in different treatment for patients with failed first TSA operation (*n* = 41).

Adjuvant Treatment	Number	Achieve GH <1 ng/ml			Achieve GH <2.5 ng/ml		
		Yes	No	*p*-value	Yes	No	*p*-value
**Second surgery**	8			1.000			1.000
**DGSA**	5	3 (60.0)	2 (40.0)		4 (80.0)	1 (20.0)	
**SGSA**	2	1 (50.0)	1 (50.0)		1 (50.0)	1 (50.0)	
**Missing**	1						
**Octreotide**	18			0.213			**0.025**
**DGSA**	13	7 (53.8)	6 (46.2)		13 (100.0)	0 (0.0)	
**SGSA**	3	0 (0.0)	3 (100.0)		1 (33.3)	2 (66.7)	
**Missing**	2						
**SRS/EXRT**	4			0.400			1.000
**DGSA**	2	1 (50.0)	1 (50.0)		1 (50.0)	1 (50.0)	
**SGSA**	2	1 (50.0)	1 (50.0)		2 (100.0)	0 (0.0)	
**Combine treatment** **^a^**	11			0.491			1.000
**DGSA**	3	1 (33.3)	2 (66.7)		2 (66.7)	1 (33.3)	
**SGSA**	8	1 (12.5)	7 (87.5)		4 (50.0)	4 (50.0)	

Fisher’s exact tests were performed. Significant results are shown in bold.

GH, growth hormone; DGSA, densely granulated somatotroph adenoma; SGSA, sparsely granulated somatotroph adenoma; SRS, stereotactic radiosurgery; EXRT, conventional external beam radiotherapy.

a Combination of surgery, SRS, and/or EXRT.

## Discussion

In this study, about 78.2% (61/78) of patients had somatotroph adenoma categorized in macroadenoma and giant pituitary adenoma. The Knosp classification of these somatotroph adenomas was grade 3 or 4. This indicates that they are hard to be removed by TSA completely. Micko et al. reported that the postoperational gross total resection ratios were 85%, 64%, and 0% and the remission rates within 2 years were 67%, 0%, and 0% for grades 3A, 3B, and 4, respectively (29). Compared with our results, there is still room for progress.

The results of this study showed that with a more stringent standard, which is GH level <1 ng/ml, about 28% of patients achieve this goal 6 months after TSA operation, and the number increased to nearly 59% at 100 months after receiving TSA. If the standard is set as GH level <2.5 ng/ml, about half of patients achieved this goal 6 months after the operation. This suggests that overall outcomes of TSA for the first TSA treatment of somatotroph adenomas are favorable. TSA has become the primary treatment for most cases of functioning pituitary adenomas (3, 5). In most cases, cure rates of more than 50% can be achieved. Campbell et al. (15) reported 26 cases of TSA for acromegaly, and the cure rate for TSA alone was approximately 60%. Hazer et al. (16) reported a cure rate of 63% in 214 patients with acromegaly treated by endoscopic transsphenoidal surgery. The cure rate of primary cases was approximately 65% (109 of 169 primary cases), and that of patients with the recurrent disease was 41% (25 of 61 recurrent patients). Another previous review study showed that the remission rate of microadenoma varied from 70% to 80%, and the remission rate for macroadenoma was 40%–60% (30). In this study, 48.8% (39/80) of the remission rate may be due to most patients with macroadenoma and giant pituitary adenoma. Another reason may be that our institution has not yet developed and performed extra-pseudocapsule dissection during the study period.

Factors associated with remission at 6 months after TSA operation are lower GH level at diagnosis and preoperation, smaller tumor size, no residual tumor, and DGSA subtype. This seems obvious logically. The interesting part is at the time of the last follow-up, only age, residual tumor, and DGSA subtype are associated with remission.

Recurrent disease, aggressive tumors, and those that do not respond to primary treatment can be difficult to treat (14, 19, 20). Treatment options include medical therapy, radiotherapy, and a second TSA surgery. Stereotactic radiosurgery is an effective option in patients with the recurrent and refractory diseases (20, 21). Medical therapy typically is treatment with somatostatin and somatostatin receptor ligands like octreotide, lanreotide, and parireotide (18). They suppress GH secretion because somatotroph adenomas, especially in DGSA, express somatostatin and dopamine receptors (18). In this study, for patients who acquired a secondary treatment, the significance only existed in SRS/EXRT method when multivariate compared with combined treatment. When further stratified with subtypes of somatotroph adenoma, octreotide had a significant effect in treating DGSA.

While TSA is considered a relatively safe procedure, it is not without complications. In this study, 3 patients died as a result of complications of the surgery. Two patients had a long history of diabetes and expired because of poor wound healing induced CSF leakage and severe CNS infection complicated with sepsis. Despite both of the patients receiving several sellar floor reconstructions including fat graft, naso-septal flap, lumbar CSF drainage, and bed rest. The other patient died due to stress ulcer-related GI bleeding after the surgery, despite the high-dose proton pump inhibitor and gastric endoscopic hemostasis procedure. Morbidities after the first operation included 24 cases of transient DI, 3 CNS infections, 10 cases of hypogonadism, 4 cases of adrenal insufficiency, 4 cases of hypothyroidism, 7 cases of CNS leakage, 3 cases of sinusitis, 3 cases of panhypopituitarism, and 1 patient who developed seizures. Not including the 3 deaths, the overall complication rate was 73.8% (59/80). Chowdhury et al. (31) prospectively studied postoperative complications in 152 consecutive patients who received TSA. The most common complications were CSF leakage (40%), DI (15%), and prolonged ventilation (15%). Other complications included bleeding/hematoma (10%), postoperative nausea/vomiting (7%), electrolyte disturbances (6%), hydrocephalus (2%), vision loss (3%), and cranial nerve palsy (1%).

There are limitations of this study. The study was retrospective, and the number of patients was relatively small. Moreover, the follow-up is not homogeneous. The time point of remission cannot be judged correctly. In the endocrinological assessment of GH, we only measured GH levels with patients fasting and did not perform an oral glucose tolerance test (OGTT) and IGF-1 level measurement. In the medical therapy, only octreotide, a first-generation somatostatin receptor ligand, was used. Since SGSA is not sensitive to this kind of compound, there will be little effect in the designed treatment. A study with more patients involved and applied subtype-specific therapeutic methods should be conducted in the future.

## Conclusions

TSA is a relatively safe and effective option for the primary treatment of GH-secreting pituitary adenomas. However, a large tumor size, high GH level at diagnosis and preoperation, and residual tumor are associated with a lower success rate. Octreotide results in good outcomes for DGSA patients, and SRS/EXRT generates good results in treating patients who receive secondary treatments when remission cannot be reached 6 months after TSA operation.

## Data Availability Statement

The original contributions presented in the study are included in the article/supplementary material. Further inquiries can be directed to the corresponding author.

## Ethics Statement

This study was approved by the Institutional Review Board of our hospital (201901259B0). Written informed consent for participation was not required for this study in accordance with the national legislation and the institutional requirements.

## Author Contributions

J-LY: conception and design, acquisition of data, analysis and interpretation of data, drafting of the manuscript, final approval of the manuscript, guarantor of integrity of the entire study, statistical analysis, definition of intellectual content, literature research, and clinical studies. M-YC: acquisition of data, analysis and interpretation of data, drafting of the manuscript, final approval of the manuscript, definition of intellectual content, and literature research. Y-LC: conception and design, acquisition of data, drafting of the manuscript, final approval of the manuscript, and clinical studies. P-WH: conception and design, acquisition of data, drafting of the manuscript, final approval of the manuscript, clinical studies, and Supervision. P-WH: conception and design, acquisition of data, drafting of the manuscript, final approval of the manuscript, and clinical studies. K-CW: conception and design, acquisition of data, drafting of the manuscript, final approval of the manuscript, and clinical studies. C-NC: conception and design, acquisition of data, drafting of the manuscript, final approval of the manuscript, clinical studies, supervision, and guarantor of integrity of the entire study.

## Funding

This research was funded by grants from Keelung Chang Gung Memorial Hospital (CMRPG2J0102, J-LY).

## Conflict of Interest

The authors declare that the research was conducted in the absence of any commercial or financial relationships that could be construed as a potential conflict of interest.

## Publisher’s Note

All claims expressed in this article are solely those of the authors and do not necessarily represent those of their affiliated organizations, or those of the publisher, the editors and the reviewers. Any product that may be evaluated in this article, or claim that may be made by its manufacturer, is not guaranteed or endorsed by the publisher.
